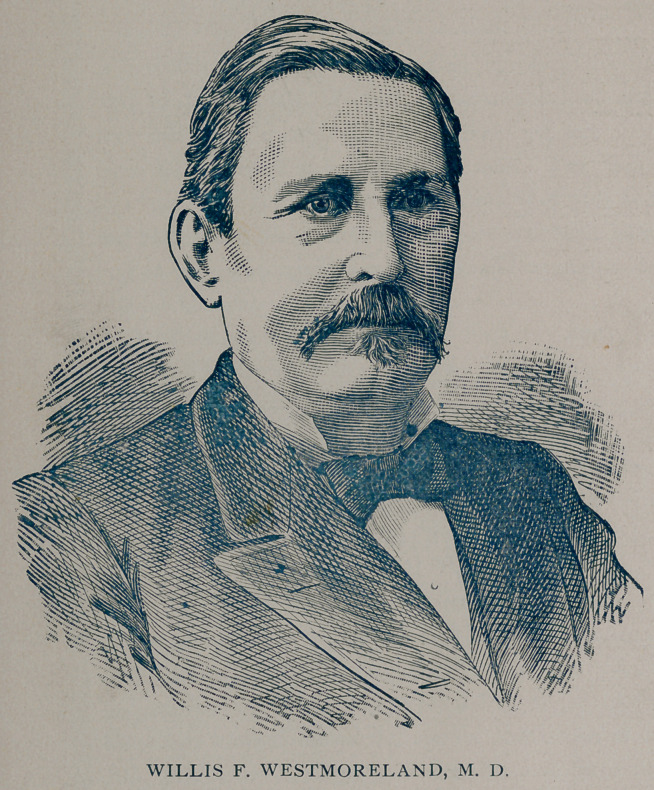# W. F. Westmoreland, M. D., Atlanta, Ga.

**Published:** 1890-07

**Authors:** 


Page(s) missing

WILLIS F. WESTMORELAND, M. D.

was chosen Professor of Surgery in the Atlanta Medical College, a position which he held and whose duties he fulfilled to the day of his death. In 1855 he founded the ATLANTA MEDICAL AND SURGICAL JOURNAL, and was actively connected with its management for more than twenty years. At the time of his death he still held a position on its editorial staff. In 1856 he again visited Europe, and spent several months in Paris perfecting himself in the art of surgery. On his return he located in Atlanta and rapidly built up a large and lucrative practice. When the war between the States came on, he offered his services to the Southern Confederacy, and they were promptly and cordially accepted. He served until the surrender at Appomatox, when he returned to Atlanta and resumed the practice of his profession. As the years rolled by his fame increased, until it extended to every State in the Union, and he came to be looked upon as the foremost surgeon of the South. For a few years before his death he was in feeble health, yet he remained at his post, and many a time arose from a bed of sickness to minister to the sufferings of others. When the hand of death was finally laid upon him, he went to his grave full of honors, and carrying with him the love, the respect and the admiration of all who knew him. He leaves a son, Dr. Willis F. Westmoreland, Jr., and a daughter, Mrs. John Rommel, of Philadelphia, to mourn a loss which to them is as irreparable as it is to the profession and to the community in which he lived.

## Figures and Tables

**Figure f1:**